# Support Vector Machine Outperforms Other Machine Learning Models in Early Diagnosis of Dengue Using Routine Clinical Data

**DOI:** 10.1155/2024/5588127

**Published:** 2024-10-14

**Authors:** Ariba Qaiser, Sobia Manzoor, Asraf Hussain Hashmi, Hasnain Javed, Anam Zafar, Javed Ashraf

**Affiliations:** ^1^Molecular Virology Lab, National University of Science and Technology (NUST), Atta-ur-Rehman School of Applied Biosciences (ASAB), Islamabad, Pakistan; ^2^Institute of Biomedical and Genetic Engineering (IBGE), KRL Hospital, Islamabad, Pakistan; ^3^Provincial Public Health Reference Lab, Punjab AIDS Control Programe, Lahore, Pakistan; ^4^Department of Pediatrics, Avicenna Medical Complex, Lahore, Pakistan; ^5^Department of Community Dentistry, Riphah International University, Islamabad, Pakistan; ^6^Institute of Dentistry, University of Eastern Finland, Kuopio, Finland

## Abstract

**Background:** There is a dire need for the establishment of active dengue surveillance to continuously detect cases, circulating serotypes, and determine the disease burden of dengue fever (DF) in the country and region. Predicting dengue PCR results using machine learning (ML) models represents a significant advancement in pre-emptive healthcare measures. This study outlines the comprehensive process of data preprocessing, model selection, and the underlying mechanisms of each algorithm employed to accurately predict dengue PCR outcomes.

**Methods:** We analyzed data from 300 suspected dengue patients in Islamabad and Rawalpindi, Pakistan, from August to October 2023. NS1 antigen ELISA, IgM and IgG antibody tests, and serotype-specific real-time polymerase chain reaction (RT-PCR) were used to detect the dengue virus (DENV). Representative PCR-positive samples were sequenced by Sanger sequencing to confirm the circulation of various dengue serotypes. Demographic information, serological test results, and hematological parameters were used as inputs to the ML models, with the dengue PCR result serving as the output to be predicted. The models used were logistic regression, XGBoost, LightGBM, random forest, support vector machine (SVM), and CatBoost.

**Results:** Of the 300 patients, 184 (61.33%) were PCR positive. Among the total positive cases detected by PCR, 9 (4.89%), 171 (92.93%), and 4 (2.17%) were infected with serotypes 1, 2, and 3, respectively. A total of 147 (79.89%) males and 37 (20.11%) females were infected, with a mean age of 33 ± 16 years. In addition, the mean platelet and leukocyte counts and the hematocrit percentages were 75,447%, 4189.02%, and 46.05%, respectively. The SVM was the best-performing ML model for predicting RT-PCR results, with 71.4% accuracy, 97.4% recall, and 71.6% precision. Hyperparameter tuning improved the recall to 100%.

**Conclusion:** Our study documents three circulating serotypes in the capital territory of Pakistan and highlights that the SVM outperformed other models, potentially serving as a valuable tool in clinical settings to aid in the rapid diagnosis of DF.

## 1. Introduction

Dengue fever (DF) is a viral infection caused by dengue virus (DENV). DENV belongs to the genus Flavivirus of family Flaviviridae. It has four serotypes (DENV-1, DENV-2, DENV-3, and DENV-4) [1]. The DENV virion is spherical in shape, enveloped with a diameter of approximately 50 nm. The genome comprises approximately 10,600 nucleotides and encodes three structural (C, prM/M, and E) and seven nonstructural proteins (NS1, NS2A, NS2B, NS3, NS4A, NS4B, and NS5) [2].

DENV is transmitted to humans via the bite of an infected *Aedes* mosquito. Each DENV serotype can cause full spectrum illness ranging from self-limited DF to severe dengue hemorrhagic fever (DHF) and dengue shock syndrome (DSS) [3]. Usually, 2–7 days long high fever is the most common symptom of dengue and other symptoms may include severe body ache including headache, bone ache, joint ache, muscle ache, nausea, mild bleeding (e.g., nose or gum), and low white cell count. Untreated DHF has a mortality rate as high as 50%, and treated DHF has a mortality rate of 2%–5%. DHF usually occurs in people who have previously been infected with a different strain of the virus [4].

At present, it affects more than 128 countries worldwide, and estimated 40% population of the world (2.5 billion) are at a risk of contracting dengue in South Asia, Pacific, and Americas. Approximately 50 million cases of dengue occur worldwide every year with 500,000 people hospitalized with severe disease. The prevalence rate has increased ∼6 fold from < 0.5 million in 2010 to over 3.34 million in 2016 [5].

Currently, no effective vaccine is available for DF. Intravenous hydration therapy is the main treatment for dengue patients with substantial vascular leakage. Climatic conditions and an unprecedented increase in trade and travel in the global village had played a major role in global and local spread of DENV. Climatic conditions have a deep impact on dengue vector life cycle and the vectors' ability to spread the disease among humans. The disease is prevented mainly by vector control [6]. The emergence of DF in Pakistan occurred in 1980s [7]. Now, it has reached an all-time high in all provinces. The recent outbreak in Islamabad and adjoining areas is the most severe affecting > 50,000 people [8]. Analysis of the outbreaks of DENV infection in Pakistan indicates a seasonal trend. All outbreaks (including the one reported here) occurred during the monsoon (rainy) season (August to November) and subsided with the onset of winter.

Pakistan is still a developing country, and many people lack proper awareness regarding DENV. The symptoms of DENV are generally common and resemble those of other diseases, leading to many cases going undiagnosed and unrecorded. Regular updates on the prevalence of DENV and its serotypes play a major role in controlling the spread of the disease. Therefore, our study aimed to illustrate the prevalence of DENV and its serotypes during the recent outbreak in the twin cities of Pakistan. Rapid diagnosis is crucial for providing timely treatment, especially in cases of DHF and DSS, where the condition becomes severe and sometimes lethal.

It is hypothesized that machine learning (ML) algorithms can accurately predict dengue PCR results using standard clinical and demographic data. Therefore, another objective of this study was to develop a predictive ML model for the RT-PCR results of DENV using only routine laboratory data obtained from the emergency department. The models were designed to learn patterns in the data that could predict the outcome variable (dengue PCR result) based on the values of the independent variables (demographic information and laboratory results).

## 2. Methods

Inclusion criteria include the following:

a. Patients experiencing febrile symptoms commonly associated with suspected dengue infection, such as high fever, headache, muscle and joint pain, nausea, and mild bleeding, were included
b. Only patients who had undergone serological tests (NS1, IgG, and IgM) and RT-PCR testing for the DENV were considered
c. To maintain geographic consistency, only residents of Islamabad and Rawalpindi were included in the study

Exclusion criteria include the following:

a. Patients with incomplete or missing laboratory data were excluded from the analysis
b. Those who tested negative for all serological markers (NS1, IgG, and IgM) and did not undergo RT-PCR testing were also excluded
c. Samples that were not processed within 1 hour of blood collection were excluded to ensure the reliability of the data

### 2.1. Subjects and Sampling

The patient cohort included a total of 300 suspected DENV patients collected in the KRL hospital, Islamabad, for serological testing ( nonstructural 1 protein [NS1], immunoglobulin [IgG] *γ*, and IgM *μ*). Patients were residents of Islamabad and Rawalpindi, Pakistan. Blood or serum samples of the patients were received from different hospitals. Plasma/serum from blood was separated within 1 h of blood collection and stored at −20°C. This study was approved by the Institute of Biomedical and Genetic Engineering Ethical Committee and was in compliance with the Helsinki Declaration, 2000.

### 2.2. RNA Extraction

Dengue RNA was extracted from 140 *μ*L of serum by using a commercially available QIAamp Viral RNA Mini Kit (Qiagen Cat. # 52906). The RNA was eluted in 60 *μ*L of TE buffer.

### 2.3. Multiplex RT-PCR Assay for Serotyping

Purified RNA of each sample (10 *μ*L) was subjected to reverse transcription and amplification by using multiplex RT-PCR kit for detection of four dengue serotypes (abTES DEN 4 qPCR kit, AITbiotech, Singapore) according to the manufacturer's instructions using the Slan 96P RT-PCR System (Sansure Biotech, Hunan, China).

### 2.4. Purification of RT-PCR Products and Direct Sequencing

PCR products were purified by the ammonium acetate (10 M) and absolute ethanol precipitation method. Of the purified PCR product, the sequencing reaction was carried out by mixing 2 *μ*L autoclaved deionized water and 4 *μ*L big dye v.3.1. 3 *μ*L purified PCR product and 1 *μ*L of forward or reverse primer making up a total volume of 10 *μ*L. PCR conditions used were 25 cycles of 96°C for 15s, 50°C for 15 s, and 60°C for 4 min and hold at 4°C. The sequence PCR products were purified using EDTA (125 mM) and ethanol precipitation. Each sample was resuspended in 10 *μ*L Hi-Di formamide. Sequencing was carried out in 3130 genetic analyzer (ABI part no. 4363785, Applied Biosystems, Foster city, CA, USA) utilizing performance optimization polymer 6 as the separation matrix. Data were collected and analyzed by ABI Sequencing Analysis Software v5.2. Sequences were serotyped by using the NCBI nonredundant database [9].

### 2.5. Data Collection

The demographic details of all 300 patients were received including gender, age, and residence. Serotypes, serological tests results, and hematological tests results of each suspected person were also recorded. The data included NS1, IGM, and IGG tests results. It also included the DENV-specific blood profile summary, having platelets (PLTs), white blood cells (WBCs) and HCT (hematocrit).

### 2.6. Statistical Analysis

The categorical data (gender, age group, and serotypes) are expressed by percentages. The continuous data (age, PLT, WBC, and HCT) were categorized into ordinal data. Then, the ordinal data were assessed by either Fisher's exact test or chi-square test. A *p* value of < 0.05 was considered statistically significant. The statistical analysis was performed using GraphPad Prism, version 9.5.1.

### 2.7. ML Methods

Statistical analyses for ML were applied through pandas, Numpy, and Scikit-learn packages. Visualization of ML results was done through Matplotlib specificity, and recall (sensitivity) and precision were used to assess the effectiveness of the proposed model.

#### 2.7.1. Outcome Variable (Dengue PCR Result)

The outcome variable was the dengue PCR result, which is a binary variable indicating the presence (positive) or absence (negative) of the DENV in the patient's blood sample. This binary outcome was used as the target variable for all predictive models, guiding the training process to accurately classify each case.

#### 2.7.2. Independent Variables

The independent variables included a range of demographic, clinical, and laboratory data points, each selected based on their potential relevance to DENV infection and their availability in the dataset.

I. Demographic information: Age groups and gender (as binary variable) were included to explore potential demographic patterns in DENV infection rates.
II. Laboratory results: NS1, IgG, and IgM were included as binary (positive and negative) variables. The variable of PLT count was further categorized into “thrombocytopenia (lower than normal range), normal, and thrombocytosis (above the normal range)” (normal range: 150,000 to 400,000 PLT/ml). The variable of WBC counts was categorized into “leukopenia (lower than normal range), normal, and leukocytosis (above the normal range)” (normal range: 4500 to 11,000 WBCs/mL). The variable of HCT percentages was categorized into “anemia (lower than normal range) and normal” (normal range: for men is 40%–54%; for women, it is 36%–48%) [10].

### 2.8. Data Preprocessing

The initial step in our analysis involved a meticulous data cleaning process. Data cleaning involved addressing missing values, outliers, and inconsistencies. Missing data were imputed using the mean for continuous variables and the mode for categorical variables, ensuring the dataset's completeness. Outliers were identified through *z*-scores and adjusted using capping methods to maintain data integrity. Categorical variables, such as gender and serological test results, were encoded using binary encoding to make them interpretable by ML algorithms. Continuous variables, including age, PLT, WBC, and HCT, were normalized using min–max scaling, aligning variable ranges to a standard scale (0-1) to promote uniformity and improve algorithm convergence rates. The dataset was split into training (70%) and testing (30%) subsets, ensuring the model learned from a representative sample while allowing unbiased performance evaluation on unseen data. This approach was critical for mitigating overfitting and enhancing the model's generalization capabilities.

### 2.9. Algorithms' Selection Logic

The selection of algorithms was guided by the need for a diverse set of models that could capture the nuances of the dataset from different perspectives. The chosen models included logistic regression, extreme gradient boosting (XGBoost), light gradient boosting machine (LightGBM), random forest, radial basis function kernel (RBF kernel) in support vector machine (SVM), and categorical boosting (CatBoost).

To assess the generalizability of the SVM model, we utilized a synthetic external dataset consisting of 200 samples. This external validation was crucial to ensure the model's applicability in different clinical settings beyond the initial study cohort. The validation process demonstrated that the SVM model maintained high accuracy and recall, indicating its effectiveness in real-world applications.

### 2.10. Hyperparameter Tuning

For circumventing the class imbalance in the dataset and enhancing the predictive accuracy of ML models for dengue PCR result prediction, a comprehensive hyperparameter tuning was processed for adjusting the algorithm parameters to improve model performance The tuning process utilized the GridSearchCV method from the scikit-learn library.

## 3. Results

### 3.1. Population Description and Statistics

**Figure 1:**
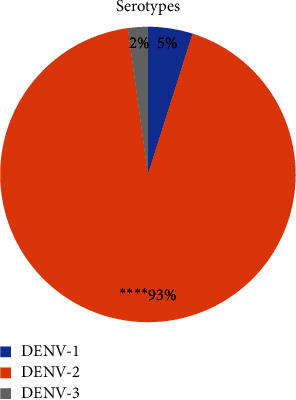
DENV serotype distribution. The pie chart represents percentages of the three serotypes of DENV infecting individuals. DENV, dengue virus; ^∗^, *p* < 0.05; ^∗∗^, *p* < 0.01; ^∗∗∗^, *p* < 0.001; ^∗∗∗∗^, *p* < 0.0001.

**Figure 2:**
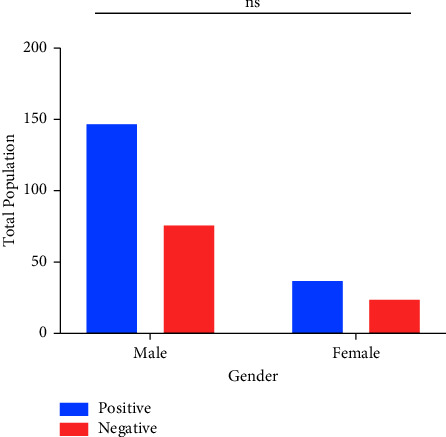
Gender characterization among DENV-infected/uninfected individuals. *Y*-axis represents the total number of individuals (both DENV positive or negative), while the *X*-axis categorizes male and female distribution. ns, nonsignificant.

Among three hundred (*n* = 300) patients, one hundred and eighty-four (*n* = 184) were PCR positive and rest were negative. Of the total positive cases detected by PCR, 9 (4.89%), 171 (92.93%), and 4 (2.17%) were infected with Serotype 1, 2, and 3, respectively. DENV-2 was found to be significantly the most prevailing with a *p* value < 0.0001 (*χ*^2^ = 441.5, d*f* = 2) (Figure 1). The total number of infected males 147 (79.89%) were observed to be higher than that of females 37 (20.11%). However, Fisher's exact test revealed no significance of infected males dominating the infected females with the odds ratio of 1.255 (95% CI; 0.7030–2.203), as shown in Figure 2.

**Figure 3:**
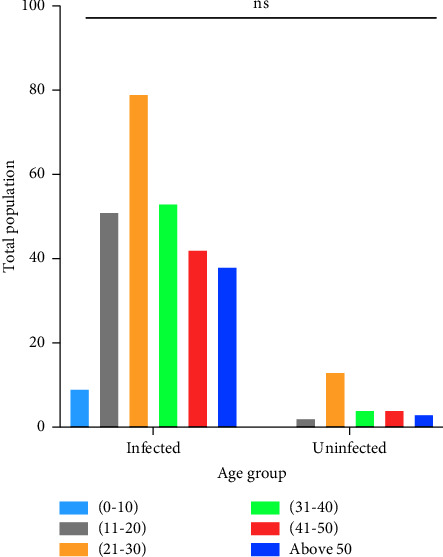
Age distribution across infected and uninfected population. *Y*-axis represents the total number of individuals, while the *X*-axis categorizes different age groups in infected and uninfected people. With 10 years gap, each age group is formed but not the last group “above 50,” which included all old aged people. ns, nonsignificant.

**Figure 4:**
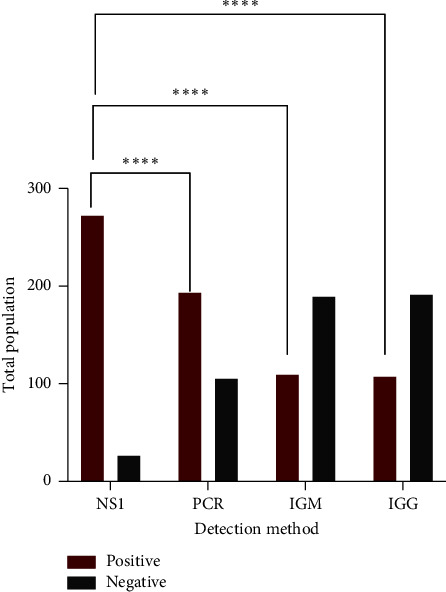
DENV detection methods. *Y*-axis represents the total number of individuals (both DENV positive or negative), while the *X*-axis categorizes detection methods as NS1 antigen ELISA, PCR, IGM, and IGG antibody tests. NS1, nonstructural 1 protein; PCR, polymerase reaction; IgG, immunoglobulin *γ*; IgM, immunoglobulin *μ*. ^∗^, *p* < 0.05; ^∗∗^, *p* < 0.01; ^∗∗∗^, *p* < 0.001; ^∗∗∗∗^, *p* < 0.0001.

**Figure 5:**
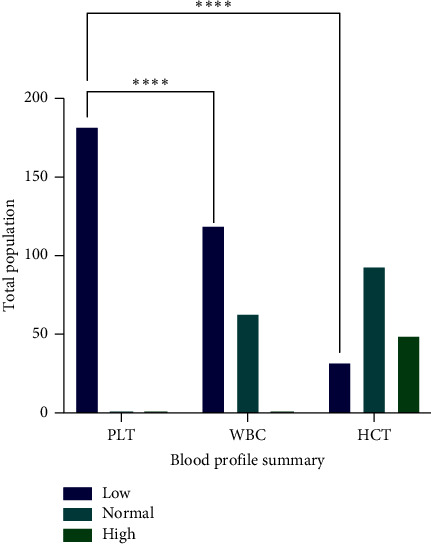
Blood profile of DENV seropositive population. The *Y*-axis represents the total seropositive individuals, while on the *X*-axis, the distribution of low, normal, and high categories of PLT, WBC, and % HCT are mentioned. Normal ranges: PLT, 150,000 to 400,000 PLT/mL; WBC, 4500 to 11,000 WBCs/mL; HCT, for men is 40%–54%; for women, it is 36%–48%. PLT, platelets; WBC, white blood cells; HCT, hematocrit. ^∗^, *p* < 0.05; ^∗∗^, *p* < 0.01; ^∗∗∗^, *p* < 0.001; ^∗∗∗∗^, *p* < 0.0001.

The numbers of infected people categorized in different age groups are as follows: 21–40 years, 79 (29.04%); 31–40 years, 53 (19.48%); 11–20 years, 51 (18.75%); 41–50 years, 42 (15.44%); above 50, 38 (13.97%); and 0–10 years, 9 (3.3%) Figure 3. Out of the 300 infected personnel, 91% (273) by NS1 antigen ELISA, 61.33% (184) by PCR, 36.67% (110) by IGM, and 36% (108) IGG antibody tests were detected. The NS1 antigen test results were highly significant with a *p* value < 0.0001 (*χ*^2^ = 253.4, d*f* = 3), Figure 4. Moreover, the hematology of the patients revealed the average of PLT, WBC, and HCT counts as 75,447 (10,800–97,000); 4189.02 (300–9300); 46.05% (12.7%–58%), respectively. The contingency test shows that a low PLT count was significantly the most common than that of WBC and HCT among the infected people with a *p* value < 0.0001 (*χ*^2^ = 279.3, d*f* = 4). Then, the low WBC count was also highly significant than that of %HCT Figure 5.

### 3.2. DENV RT-PCR Results' Prediction by ML Model

**Figure 6:**
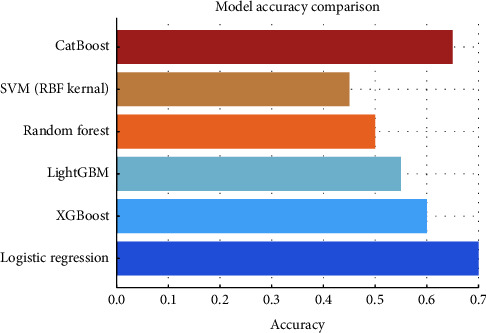
Visual comparison of various classification algorithms based on accuracy.

In the context of the dengue PCR result variable in our dataset, the ratio of negative to positive results is approximately 1:1.86, meaning the minority class (negative results) makes up about 35% of the data. The results obtained by different ML algorithms are described in Table 1 and Figure 6.

After evaluating various ML models, the SVM with a radial basis function (RBF) kernel was identified as the best-performing model. This choice was based on its superior recall and accuracy metrics, demonstrating high sensitivity to detect dengue PCR positives. Also, we conducted this validation using a synthetic external dataset (details in Table 2) of 200 samples, which confirmed the model's robustness and generalization capabilities.

**Figure 7:**
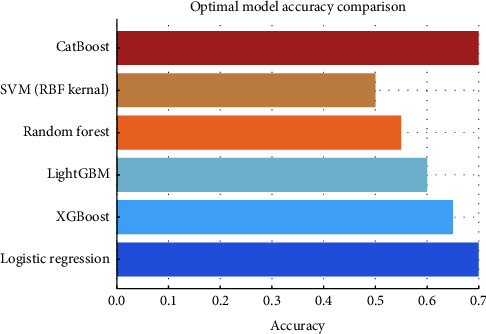
Visual comparison of evaluation metrics of various classification algorithms based on hyperparameter tuning.

**Figure 8:**
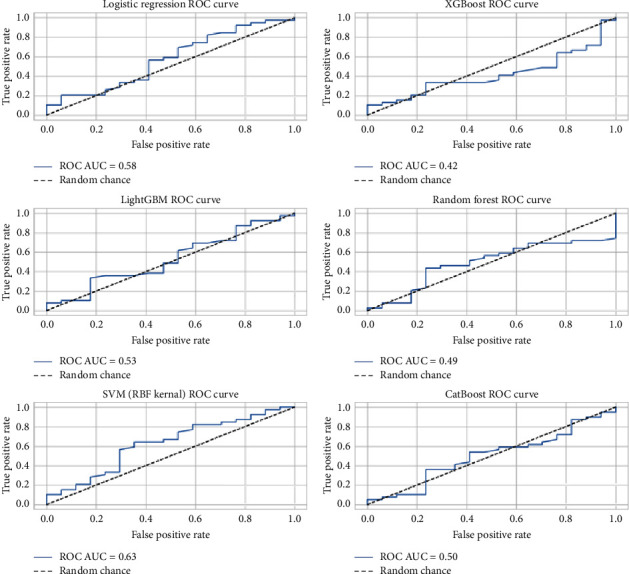
Visual comparison of ROC AUC of various classification algorithm.

After the hyperparameter tuning, the models demonstrated varied improvements in accuracy, recall, and precision metrics, indicative of their enhanced ability to predict dengue PCR results accurately. The visualization of these metrics post-tuning provided a clear comparative analysis and declared the logistic regression, LightGBM, and SVM (RBF Kernel) the most effective models for this specific application as described in Table 3 and Figures 7 and 8.

## 4. Discussion

DF is the most common vector-borne disease worldwide. Tropical and subtropical regions frequently experience its outbreaks during the monsoon season due to increase in mosquitoes [11]. Pakistan is also characterized as dengue endemic region, but the actual number of infections remains unclear due to lack of proper diagnosis and surveillance facilities. The majority of cases were asymptomatic or mild infections and are self-managed, so the actual number of dengue cases is underestimated. Most of the cases are self-managed because of asymptomatic conditions or mild symptoms of febrile illnesses [12].

This retrospective study aims to report the dengue seropositive cases in the cities of Islamabad and Rawalpindi of Pakistan, since July 2023 to October 2023. The data obtained from clinical suspects with common symptoms of fever, myalgia, retro-orbital pain, and headache tested for DENV detection by PCR. The total number of tests performed was 300, out of which, 184 came positive contributing to 58.48% of infection. Serotype1, 2, and 3 were detected, and the most prevalent was DENV-2 among the total infected individuals (*p* < 0.001). The leading DENV-2 with 171 (92.93%), followed by DENV-1 with 9 (4.89%) and DENV-3 with 4 (2.17%), individuals. This finding correlates with previous studies by Khan et al. [13, 14].

Gender distribution among infected and uninfected individuals revealed a higher number of males over females, i.e., 147 (79.89%) and 37 (20.11%), respectively, *p* = 0.45. The excess number of infected males by dengue has been also observed in other regions of South Asia. The literature suggests that greater exposure of males to the mosquitoes (vectors) due to spending more time outdoors could be a possible explanation to this observation [15].

The results of different detection methods show the highest number of DENV detection was by NS1 antigen test, i.e., 91% (273) (*p* < 0.0001) followed by PCR; 61.33% (184), IGM; 36.67% (110), and IGG; 36% (108) antibody tests. The NS1 serological test is rapid and accurate with overall sensitivity of 91% approximately for all the serotypes. The nonstructural protein of DENV can be detected in blood from the first day of infection till the maximum of 10 days [16]. On the other hand, PCR is the gold standard of detecting DENV genomic RNA during the early stages of infection. PCR-based detection can not only differentiate DENV from other flaviviruses but also it offers serotype-specific recognition [17].

The hematology revealed clinical conditions of thrombocytopenia, leukocytopenia, and anemia of the suspected population. It was determined by the statistical analysis that thrombocytopenia was significantly most observed clinical characteristics of DENV. In addition, leukocytopenia was also significantly more observed than that of anemia. Previous research studies by Ananda Rao et al. also considered these two clinical manifestation as prognoses for DENV with common DF-like symptoms [18, 19].

The most common age group infected by DENV was the “21–30 years” old, i.e., 79 (29.04%). A similar study by Qamash et al. also reported the same age group of infected young adults to be dominating the others [20]. The age group of “0–10 years” reported to be the least number of infected individuals as these young children who are mostly under immense care and protection from insects' bites.

In discussing the results obtained from the application of various ML algorithms to dengue PCR result prediction, it is pertinent to consider several factors impacting models' performances in the current study. Firstly, the challenge of a small sample size (*n* = 300) is a significant concern in ML research, as it can restrict the model's ability to generalize effectively. A study by Vabalas et al. highlights the influence of small sample sizes on ML algorithm validation, emphasizing the potential for overfitting and reduced predictive performance [21]. This aligns with the findings in our study, where, despite the small dataset (in ML context), the models achieved satisfactory performance, suggesting robustness in the face of limited data.

Secondly, the challenge of data imbalance, where negative cases made up around 35% of our dataset, presented another hurdle. The research indicates that class imbalance can heavily influence the performance of ML models, often resulting in biased predictions that favor the majority class [22]. To address this issue, we applied hyperparameter tuning and carefully selected models, demonstrating a thoughtful approach to minimize bias and improve the reliability of our results.

Lastly, the remarkable achievement of 100% recall in two of the models post-tuning underscores the importance of this metric in disease prediction contexts. High recall is crucial in medical diagnostics, where failing to identify true positives (i.e., actual disease cases) can have severe implications. This aspect of the results highlights the potential of ML models, even with imbalanced data, to achieve significant outcomes in terms of sensitivity to disease presence, aligning with the broader goal of enhancing diagnostic tools through advanced analytics.

These considerations collectively underscore the nuanced interpretation of the study's results, demonstrating that despite inherent data challenges, strategic model selection and tuning can yield highly effective tools for disease prediction. The insights drawn from this analysis contribute to the ongoing discourse on leveraging ML in healthcare, particularly in optimizing diagnostics in the face of data limitations and imbalance. The optimal parameters of artificial intelligence, logistic regression, LightGBM, and SVM (RBF kernel) predicted RT-PCR results for DENV, with accuracy of 69.64% and 71.43%, respectively. The identification of these algorithms as effective predictors aligns with findings from other studies, such as those by Chandrakantha and Salim et al. [23, 24], who also dealt with smaller sample sizes and dataset imbalances. These studies reinforce the robustness of ML-based approaches for dengue detection. For example, Mayrose et al. achieved high accuracy (93.62%) in detecting dengue from blood smear images using SVM and decision tree models. Their focus on feature extraction from PLT and lymphocyte characteristics revealed that morphological and textural features play a crucial role in accurate dengue detection from peripheral blood smears. In addition, their use of a pretrained MobileNetV2 to extract deep features from lymphocyte nuclei highlights the potential of AI to enhance traditional clinical diagnostics [25]. Compared to our study, their reliance on image-based data resulted in slightly higher performance metrics, suggesting that integrating imaging data with clinical records could further improve dengue prediction models.

Similarly, Guo et al. developed a dengue forecasting model using ML that incorporated climate factors and search query data. Their SVR model significantly outperformed others in predicting dengue outbreaks across different Chinese provinces. By incorporating external data sources, such as meteorological factors and RT search data, Guo et al. were able to improve both predictive accuracy and timeliness. In contrast, our study focused solely on clinical and laboratory data, which may account for differences in predictive performance. Incorporating external environmental data in future iterations of our model could lead to greater accuracy, especially for early outbreak detection [26].

SVM emerged as the superior model in this study due to its ability to handle nonlinear relationships in high-dimensional spaces effectively. SVM's performance, particularly with the RBF kernel, demonstrated robust generalization capabilities, achieving 71.4% accuracy and 97.4% recall before tuning and 100% recall after hyperparameter optimization. This aligns with SVM's known strength in binary classification tasks with complex feature interactions [27]. The model's resilience to overfitting, even with the study's relatively small dataset and class imbalance, further underscores its suitability for early dengue diagnosis using routine clinical data.

## 5. Conclusion and Limitation

Decisively, the assessment of a patient's infection status, and the risk of a fatal disease outcome during the course of the disease can be accurately predicted through ML models ahead of DENV PCR results. Moreover, this study provides a comprehensive report on DENV outbreak with its serotype-2 prevalence in the twin cities of Pakistan. It also highlights males in the age Group 21–30 as the most vulnerable population to DENV infection. The importance of serological testing as a means of a rapid diagnosis has also been reported. Thrombocytopenia and leukocytopenia have been characterized as prognostic markers to support the serological and molecular based detection.

Limited number of samples leads to the major limitation of this study. Therefore, further epidemiological studies should be frequently performed to report the exact figure of infection, severity, and fatality caused by DENV. Also, with greater sample size, future research could explore the impact of feature engineering, alternative tuning strategies, and the integration of ensemble methods to further enhance predictive accuracy. It will help authorities to increase surveillance of DENV control and ultimately reduce chances of further endemics and epidemics.

## Figures and Tables

**Table 1:** Evaluation metrics for various classification algorithms.

Model	Accuracy	Recall	Precision
Logistic regression	0.70	0.97	0.70
XGBoost	0.50	0.67	0.63
LightGBM	0.66	0.85	0.72
Random forest	0.52	0.67	0.65
SVM (RBF kernel)	0.71	0.97	0.72
CatBoost	0.64	0.87	0.69

**Table 2:** Evaluation metrics for synthetic external dataset.

Validation	
Accuracy	75%
Precision	0.76
Recall	0.80
*F*1 score	0.78
ROC AUC score	0.88

**Table 3:** Comparison of evaluation metrics of various classification algorithms based on hyperparameter tuning.

Model	Accuracy	Recall	Precision	*F*1 score	ROC AUC curve score
Logistic regression	0.70	1.00	0.70	0.81	0.57
XGBoost	0.68	0.97	0.69	0.65	0.42
LightGBM	0.70	1.00	0.70	0.78	0.53
Random forest	0.68	0.97	0.69	0.68	0.49
SVM (RBF kernel)	0.70	1.00	0.70	0.83	0.62
CatBoost	0.68	0.97	0.69	0.77	0.50

## Data Availability

The data that support the findings of this study are available from the corresponding author on request. The data are not publicly available due to privacy or ethical restrictions.
